# Diversity of Hard Ticks (Acari: Ixodidae) Fauna on Green Habitats of Urban Areas in Eastern Croatia

**DOI:** 10.3390/pathogens14101010

**Published:** 2025-10-07

**Authors:** Stjepan Krčmar, Petra Matak, Lora Krčmar, Kristina Nikolić

**Affiliations:** Department of Biology, Josip Juraj Strossmayer University of Osijek, Cara Hadrijana 8/A, 31000 Osijek, Croatia; matakpetra1@gmail.com (P.M.); lora.krcmar@biologija.unios.hr (L.K.); kristina.nikolic@biologija.unios.hr (K.N.)

**Keywords:** hard ticks, Ixodidae, urban areas, Osijek, Vinkovci, Vukovar, Croatia

## Abstract

Hard ticks (Acari: Ixodidae) are vectors of numerous pathogenic microorganisms in humans and animals. In Croatia, very few studies have been conducted on the diversity of hard tick fauna in urban green areas. Therefore, the aim of this study was to investigate the diversity and seasonal dynamics of hard tick fauna in three eastern Croatian cities. Three species of ticks were recorded in Osijek and Vukovar, while six species were recorded in the area of the city of Vinkovci. In total, six species were collected during this study, and together with an earlier record of *Ixodes canisuga*, seven species are now known from urban green habitats in this region. The most abundant species was *I. ricinus* (82.06%), followed by *R. sanguineus* s.l. (9.03%), *H. concinna* (6.51%), *D. reticulatus* (1.26%), *I. hexagonus* (0.91%), *D. marginatus* (0.11%), and *I. canisuga* (0.11%). The detection of *R. sanguineus* s.l. in Vinkovci and Vukovar represents the first record of this species in eastern Croatia. In Osijek and Vinkovci, *I. ricinus* was the most abundant species. This tick is the primary vector of Lyme disease and tick-borne encephalitis, the two most common tick-borne diseases affecting humans in eastern Croatia. In Osijek and Vukovar, peak abundance of ticks was recorded in May, while in Vinkovci in March. Given that the species documented here represent 30.43% of all tick species recorded in Croatia, their presence in urban areas highlights the potential public health risk associated with ticks in these environments.

## 1. Introduction

Hard ticks (Acari: Ixodidae) are ubiquitous vectors of numerous animal and human pathogens [1]. Globally, they are considered the primary vectors of animal diseases and the second most important vectors of human diseases [2,3]. They are obligatory hematophagous ectoparasites on numerous wild vertebrate species, livestock, pets and humans [4]. Ticks with a wide geographic distribution, diverse host range, and the ability to transmit multi-host pathogens, play an important role in shaping wild host population dynamics and in the epidemiology of pathogens [5]. It is estimated that approximately 10% of all known ticks are vectors of pathogens such as bacteria, viruses, and protozoa [6]. Generally, ticks and tick-borne diseases are a growing burden worldwide, with re-emerging diseases affecting human and animal health [7,8]. Factors behind this increase include the detection of new pathogens and the emergence of epidemics in areas previously free of them, often driven by local conditions [8]. Tick distribution, seasonality, and habitat preferences are strongly influenced by climatic conditions, such as mild winters and the early arrival of warm weather in the northern hemisphere [9,10,11]. Many human actions such as land-use change, abandonment of agricultural areas, and deforestation are considered as the main reasons for epidemics of tick-borne pathogens in both animals and humans [8]. Furthermore, ticks are easily spread around the world through international pet trade, frequent tourist travel with pets, through the transport of other domestic animals, or via bird and wild animal migration [12,13,14]. For these reasons, knowledge of tick distribution across Europe and beyond is of medical and veterinary importance, providing an essential basis for early detection and monitoring of tick-borne pathogens [15]. Most studies of tick distribution focus on natural environments and the associated risk of pathogen transmission [16]. However, given their important vector role, research on ticks in urban areas across Europe also began more than three decades ago [17]. The high mobility of urban populations and frequent contact of humans and their pets with wild animals, can contribute to changing epidemiological epizootiological conditions in cities [16,18]. Green urban areas (urban forests, parks, open green spaces for recreation, and hedgerows) contain diverse plant species for possible migration of wildlife from surrounding rural habitats [19]. These green areas also ensure suitable habitats for biodiversity in urban areas as well as a place for tick hosts, from small- to medium-sized mammals and larger mammals, birds, and pets (dogs and cats) [7,19]. Consequently, interactions between wild and urban fauna are frequent, particularly in expanding cities where suburban housing often borders forests [18]. Ticks have been recorded in urban areas such as parks or recreational areas across many European cities [18], including Stockholm, Bath, Bristol, and Southampton [19,20]. In Central Europe, urban tick populations have been reported in Berlin, Hanover, Budapest, Ostrava, Zielona Góra, and Warsaw [21,22,23,24,25]. In Croatia, most studies on tick diversity and vector potential have focused on natural habitats. Urban green areas have been less studied and rarely monitored, with the notable exception of Zagreb, which is a well-known natural focus of Lyme borreliosis and tick-borne encephalitis [26]. The present study therefore aimed to document the diversity of hard ticks in urban green habitats of three eastern Croatian cities, analyze the abundance of recorded species, assess their vector potential, and examine seasonal dynamics.

## 2. Materials and Methods

### 2.1. Study Area

The study was conducted in the urban green habitats of three cities in eastern Croatia. Osijek is located in Osijek-Baranja County, while Vinkovci and Vukovar are in Vukovar-Srijem County. Both counties lie within the Pannonian region, a predominantly lowland plain in northeastern Croatia [27]. The climate is temperate continental (Köppen Cfb), with average annual temperatures of 10–11 °C and precipitation ranging from 600 to 800 mm. The main landscape values of these two counties are the agrarian landscape with oak forest complexes beyond the reach of flood water and lowland floodplain forests [27].

### 2.2. Sampling of Hard Ticks in Green Habitats of the City of Osijek

**Figure 1:**
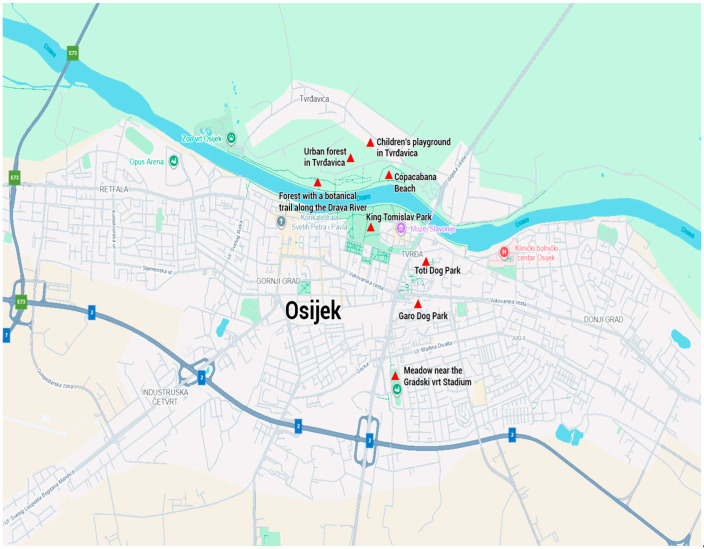
Sampling localities of hard ticks in green habitats of the city of Osijek.

The city of Osijek, located on the right and partly on the left bank of the Drava River, has the greatest number of green spaces and parks in the country; 17 in total [28]. Tick sampling was conducted at eight localities. Four sites are located on the left bank of the Drava River and four on the right bank. The first locality is an urban poplar (*Populus* sp.) forest in Tvrđavica (cadastral parcel no. 619, Tvrđavica-Podravlje) near the Osijek Zoo (45°34′69″ N, 18°41′21″ E). The second locality is a children’s playground in Tvrđavica (45°34′16″ N, 18°40′58″ E), overgrown with grasses that are regularly mowed. The third locality is the Copacabana beach (45°33′55″ N, 18°41′50″ E), the edges of which are covered with shrubby vegetation and poplar trees. The fourth locality is a forest with a botanical trail situated between Copacabana beach and the White Bridge along the Drava River (cadastral parcel no. 617; 45°33′49″ N, 18°41′8″ E). The fifth locality is King Tomislav Park (45°33′40″ N, 18°41′29″ E), a Monument of Park Architecture with 83 taxa of shrubs and trees. The sixth locality is a mesophilic meadow near the Gradski vrt stadium (45°32′35″ N, 18°41′36″ E). The seventh locality is the Garo dog park (45°33′12″ N, 18°41′53″ E), and the eight locality is the Toti dog park (45°33′29″ N, 18°41′55″ E) which are regularly mowed and frequently visited by dogs and their owners (Figure 1).

### 2.3. Sampling of Hard Ticks in Green Habitats of the City of Vinkovci

**Figure 2:**
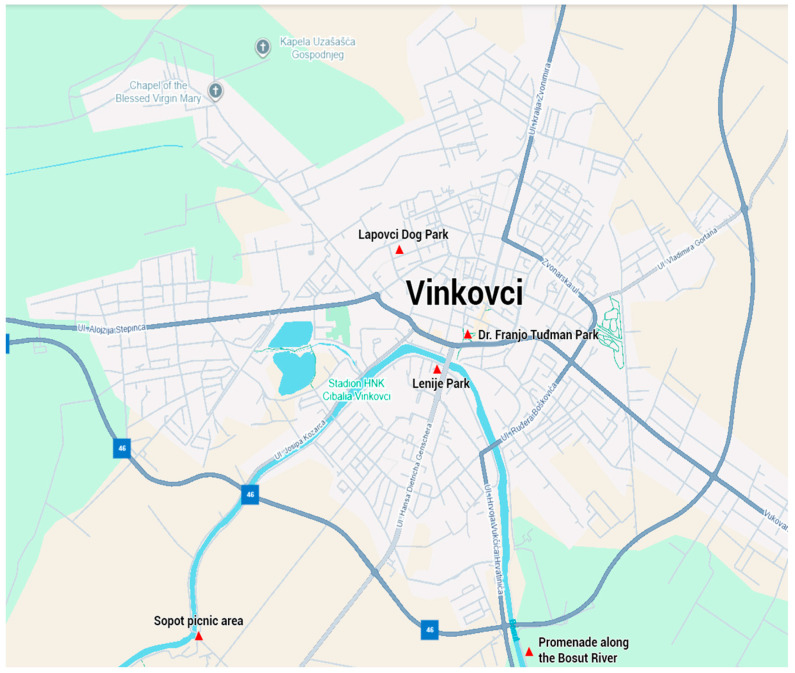
Sampling localities of hard ticks in green habitats of the city of Vinkovci.

The city of Vinkovci lies on the right bank of the Bosut River, one of the main water bodies of the area [28]. The city and its surroundings are predominantly flat, with extensive arable land and large pedunculate oak (*Quercus robur* L.) forests. Tick sampling was carried out at five localities. The first locality is Dr. Franjo Tuđman park (45°17′13″ N, 18°48′7″ E) located in the city center of Vinkovci. The park is dominated by grassy areas that are regularly maintained and contains woody vegetation throughout. The second locality is Park Lenije (45°17′01″ N, 18°47′51″ E), a Monument of Park Architecture covering about 4 ha with grassy areas and woody species (around 770 trees). The third locality is a dog park located in the Lapovci city district (45°17′48″ N, 18°47′45″ E), a fenced and regularly maintained park surrounded by residential buildings. The fourth is the Bosut River promenade (45°13′52″ N, 18°48′39″ E), a recreational area with meadows, a children’s park, gazebos, and riparian vegetation. The fifth site is the Sopot picnic area (45°15′57″ N, 18°46′19″ E) on the city outskirts, dominated by meadows and forest edges, often visited by people with dogs (Figure 2).

### 2.4. Sampling of Hard Ticks in Green Habitats of the City of Vukovar

**Figure 3:**
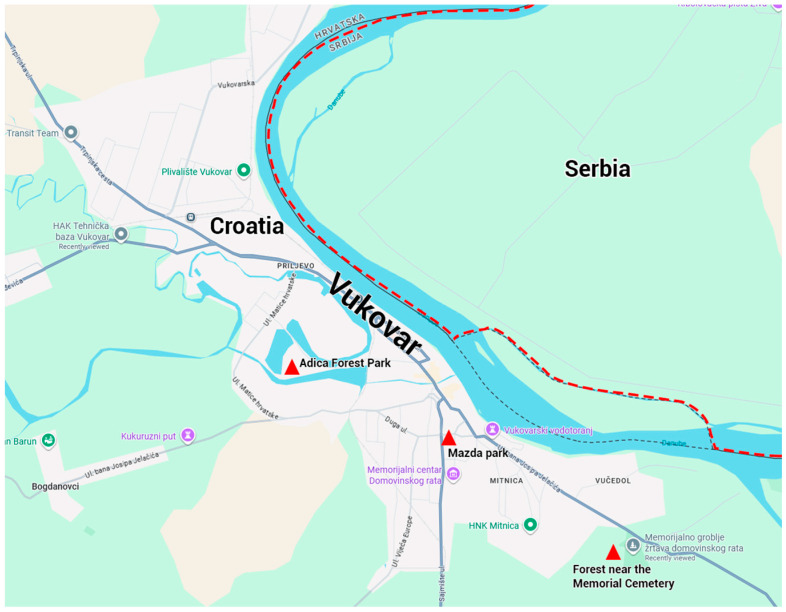
Sampling localities of hard ticks in green habitats of the city of Vukovar.

The city of Vukovar is located at the confluence of the Vuka and Danube rivers. Tick sampling was conducted at three sites. The first is Adica Forest Park (45°21′10″ N, 18°58′21″ E), about 2 km from the city center, consisting of mixed trees with numerous footpaths, bordered by marshy areas of the Vuka River. The second locality is the Mazda Park (45°20′38″ N, 19°0′13″ E) with many deciduous and coniferous tree species. The third locality is a mixed acacia (*Robinia pseudoacacia* L.) forest near the Memorial Cemetery of the Victims of Homeland War (MCVHW), (45°18′47″ N, 19°01′06″ E) (Figure 3).

### 2.5. Tick Collection, Identification, and Analysis

From March to the end of May 2023, tick sampling was conducted in Vinkovci at five green habitats, while in Osijek sampling was carried out at eight habitats from mid-March to the end of August 2025. In Vukovar, ticks were sampled during the spring months, from March to mid-May 2025. Tick samples were collected using the flag dragging method in diverse green habitats (urban forest, green spaces of recreational centers, parks, playgrounds, dog parks, the Bosut and Drava riverbanks, picnic sites), and manually from dogs during or after a walk. The majority of ticks in Vinkovci and Vukovar were obtained from dogs. All specimens were put into plastic vials and preserved in guanidine thiocyanate or 96% EtOH. Identification of species and sexes was performed using a Carl Zeiss Jena stereo-microscope (×40 magnification), according to available identification keys and illustrations [29,30,31,32]. A total of 120 ticks collected in Vinkovci were sent to the Croatian Veterinary Institute in Zagreb (Croatia) for pathogen analysis, while 622 ticks collected in 2025 were stored in the fridge at 4 °C in the Department of Biology, Josip Juraj Strossmayer University of Osijek and sent to Institute of Medical Microbiology University of Hospital Bonn (Germany) for future pathogen isolation. Differences in tick abundance among green habitats were tested using the chi-square test, with p values < 0.05 considered statistically significant. Quantitative analysis of the diversity of tick fauna between green habitats was performed by Shannon–Wiener and Simpson index [33]. Tick images were taken with a Dino-Lite digital microscope under 40× to 70× magnification.

## 3. Results

**Figure 4:**
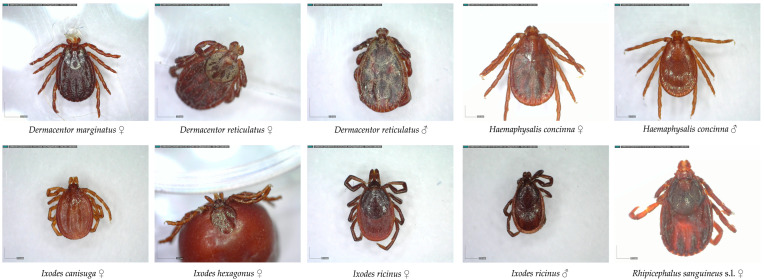
Identified hard tick species in urban green areas in Osijek-Baranja and Vukovar-Srijem County.

The collected tick specimens were identified based on morphological characteristics. Together with an earlier record of *Ixodes canisuga* [34], the following seven species are now known from urban green habitats in Osijek-Baranja and Vukovar-Srijem County: *Dermacentor marginatus* (Sulzer, 1776), *Dermacentor reticulatus* (Fabricius, 1794), *Haemaphysalis concinna* (Koch, 1844), *Ixodes canisuga* Johnston, 1849, *Ixodes hexagonus* (Leach, 1815), *Ixodes ricinus* (Linnaeus, 1758)*,* and *Rhipicephalus sanguineus* s.l. (Latreille, 1806) (Figure 4).

Only genus *Ixodes* was represented with three species, followed by the genera *Dermacentor* with two, and *Haemaphysalis* and *Rhipicephalus*, each with one species. A total of 875 ticks were collected, predominantly from Osijek (626), followed by Vinkovci (194), and Vukovar (55). The most abundant species was *I. ricinus* (82.06%), followed by *R. sanguineus* s.l. (9.03%), *H. concinna* (6.51%), *D. reticulatus* (1.26%), *I. hexagonus* (0.91%), *I. canisuga* and *D. marginatus* each with (0.11%) (Table 1).

Adult ticks predominated (82.62%), while nymphs comprised 17.37%, with a similar pattern across all cities. A total of 503 adults and 123 nymphs were recorded in Osijek, 167 adults and 27 nymphs were recorded in Vinkovci, and 53 adults and 2 nymphs were recorded in Vukovar. Vinkovci had the highest species diversity (six species), followed by Osijek and Vukovar with three species each. The finding of an adult specimen of *R. sanguineus* s.l. in the green habitats in Vinkovci and Vukovar represents the first record of this species for Eastern Croatia. Recording of *D. reticulatus* represents the first record of this species in the city of Osijek. In Osijek, 99.36% of ticks were collected along the Drava River, mainly in the urban poplar forest (74.28%) (Table 2).

In Osijek, only Toti dog park and King Tomislav Park yielded no ticks. In Vukovar, 5.45% of ticks were collected in the Adica Forest Park (Table 3).

A similar proportion of collected ticks was recorded in green habitats within the city of Vinkovci with 1.54% in Dr. Franjo Tuđman park (Table 4).

**Figure 5:**
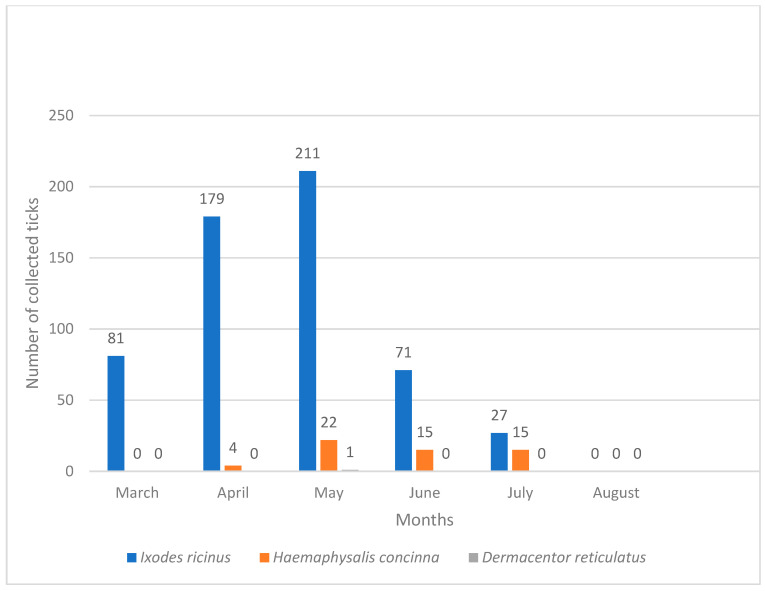
Seasonal dynamics of hard ticks in the city of Osijek.

**Figure 6:**
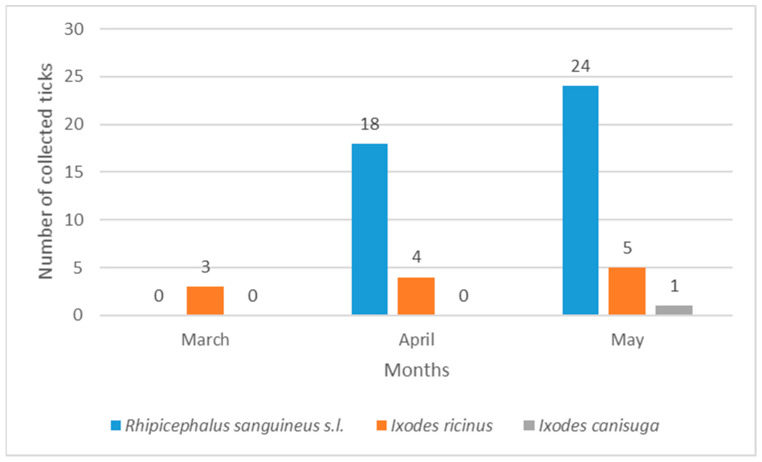
Seasonal dynamics of hard ticks in the city of Vukovar.

**Figure 7:**
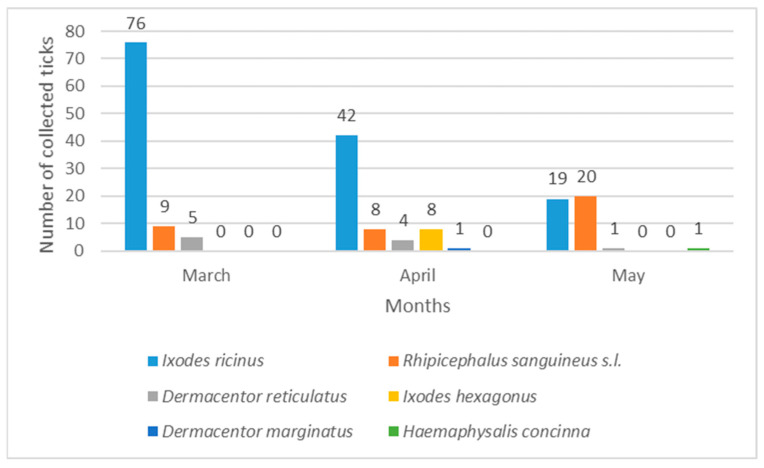
Seasonal dynamics of hard ticks in the city of Vinkovci.

Indoor ticks in Vinkovci and Vukovar belonged to *I. ricinus* and *R. sanguineus* s.l., while in Osijek only *I. ricinus* was found. Tick numbers differed significantly between habitats in Osijek (χ^2^ = 1568.4, *p* < 0.05), Vinkovci (χ^2^ = 60.87, *p* < 0.05), and Vukovar (χ^2^ = 47.24, *p* < 0.05). The lower values of H = 0.070 and S = 0.038 in Osijek, as well as H = 0.108 and S = 0.060 in Vukovar, indicate lower tick diversity, while the higher values of H = 0.604 and S = 0.328 for Vinkovci indicate higher tick diversity. Seasonally, 85.37% of ticks were collected in spring (March–May) and 14.62% in summer (June–August). In Osijek, peak abundance was in May (37.38%) and the lowest in August (0.0%) (Figure 5). In Vukovar, most ticks were collected in May (54.54%), while in Vinkovci, March had the highest abundance (46.39%) (Figure 6 and Figure 7).

*I. ricinus* was consistently the most abundant species in Osijek throughout the study (Figure 5). In Vinkovci, *I. ricinus* was also most abundant species, primarily in March and April (Figure 7). *Rhipicephalus sanguineus* s.l. dominated Vukovar as the most abundant species in April and May (Figure 6).

## 4. Discussion

### 4.1. Tick Diversity in Urban Areas of the Studied Cities

Research on hard tick diversity in urban environments in Croatia is limited, with continuous monitoring mainly in Zagreb due to its status as a natural focus of Lyme borreliosis and tick-borne encephalitis [26]. The first sampling in Osijek was conducted in 2019 [35]. In 2025, five of the eight localities sampled were the same as in 2019. A similar number of ticks were collected in both studies (664 in 2019 vs. 626 in 2025), but the proportion of *I. ricinus* increased from 74.25% to 90.89%, while *H. concinna* decreased from 25.75% to 8.94%. The dominance of *I. ricinus* is consistent with its widespread distribution in Europe [7,36]. Unlike 2019, when nymphs and larvae predominated in Osijek, adults were the majority in the present study. A similar predominance of nymphs and larvae was recorded in southern England (Bath, Bristol, and Southampton) [19]. The highest tick abundance in Osijek was recorded in the urban poplar forest along the Drava River (74.28%), similar to 2019, while green habitats within the city yielded very few ticks (0.63%). Limiting factors for tick abundance in urban areas caused by growing urbanization include reduced availability of medium- to large-sized hosts and low relative humidity during periods when tick life stage is not connected with its hosts [7,37]. These limiting factors are probably the reason for the relatively small number of ticks collected in green habitats within the studied cities of eastern Croatia. The opposite occurs in urban forests, where tick density increases with the degree of connectivity with potential host populations [37], explaining the high proportion of ticks in Osijek’s urban forest. In this urban poplar forest on the left bank of the Drava River in Osijek, various mammals such as roe deer, wild rabbits, foxes, and martens have been observed as potential hosts for ticks. Three tick species were recorded in Osijek and Vukovar only, while six were found in Vinkovci, likely due to differences in sampling methods: flag dragging alone in Osijek versus a combination of flag dragging and collection from dogs in Vinkovci. Similar findings have been reported in German cities (Munich, Regensburg, Ingolstadt, Augsburg, and Berg), where species diversity increased when multiple collection methods were used [18,38]. Six tick species were recorded in the Bosut River and Sopot picnic area habitats, reflecting connectivity with natural habitats and wildlife movement. Conversely, only two species were found in Vinkovci’s city center (Table 4) and in the city center of Vukovar (Table 3), comparable to species numbers found in similar urban habitats elsewhere in Croatia and Europe, such as Čakovec, Đakovo, and Belgrade [39,40,41]. In green areas of Helsinki, Bath, Bristol, and Southampton, one species of ixodid tick was recorded, while four were found in Hanover and ten were found in Berlin [19,23,24,42]. Agroecological programs promoting habitat connectivity may further influence tick diversity and distribution [43], as seen with urban hedgehog populations maintaining stable tick populations [44]. Low diversity index values were recorded in this study for most of the surveyed cities. However, in Vinkovci, the Shannon and Simpson indices indicate notably higher tick diversity. The generally low diversity values can be partially explained by the spatial limitation and fragmentation of urban green areas, which reduce contact with surrounding natural habitats. This limited ecological connectivity likely restricts the movement of tick hosts and the establishment of a broader range of tick species, resulting in fewer species being recorded in more isolated urban environments.

### 4.2. Rhipicephalus sanguineus *s.l.*

Of particular note, *R. sanguineus* s.l. was recorded for the first time in eastern Croatia and was the most abundant tick species in Vukovar and the second most abundant species in Vinkovci (Table 1). This species prefers dogs and human dwellings and can establish local populations year-round [18,45]. Its occurrence in four of the five Vinkovci habitats (Table 4), all frequented by dogs, suggests potential establishment. In Vukovar, it has only been recorded in Mazda Park, and all specimens were collected from one dog. Previously, *R. sanguineus* s.l. in Croatia was mostly confined to the Mediterranean region and cities of Pula, Zadar, and Dubrovnik [39]. Its introduction to Vinkovci and Vukovar may have been via pets, resulting in local colonization, since it was absent in a prior study of hard tick fauna across 48 localities in eastern Croatia [34]. The introduction of *R. sanguineus* s.l. ticks into new areas has in many cases been followed by a rapid infestation of dogs and the development of local tick colonies [18]. *Rhipicephalus sanguineus* s.l. is the most important tick from a veterinary point of view, as it is a vector of disease agents such as *Coxiella burneti*, *Ehrlichia canis*, *Rickettsia conorii*, *Rickettsia rickettsii*, and *Rickettsia massiliae* [45,46]. Domestic dogs are the main host of *R. sanguineus* s.l. in both urban and rural areas, where tick infestation is often higher among urban dogs than in rural ones [45]. Recently, several new foci of *R. sanguineus* s.l. have been found in neighboring Hungary, as well as pathogens associated with this species [46]. Global warming may contribute to the establishment of populations of these ticks in areas that were previously free of them [45] and to the shift to a more thermophilic tick fauna in this part of Europe [46]. That may explain records of this species in the area of eastern Croatia.

### 4.3. Seasonal Dynamics of Hard Ticks

Seasonally, *I. ricinus* was most abundant, most likely due to its high adaptability [47], with a peak in May in Osijek and Vukovar, consistent with previous regional studies [48]. In Vinkovci, *I. ricinus* abundance decreased from March to May, similar to patterns observed in Budapest [49]. *Haemaphysalis concinna* was active from mid-April to late July, peaking in May, slightly earlier than its usual Central European peak in June [50]. *Rhipicephalus sanguineus* s.l. showed peak activity in May, whereas in Goiânia, State of Goiás (Brazil), adults exhibited four activity peaks per year [51]. *D. reticulatus* peaked in March, consistent with patterns reported in Budapest and in earlier studies from eastern Croatia [34,49]. In this study, *D. reticulatus* was the fourth most abundant species, despite being the second most common tick in many parts of Europe [14,52,53]. *Dermacentor reticulatus* is well adapted to colder environments and seasons and hence is common in autumn and winter months [52]. This study does not extend into autumn and winter months, which could explain the lower number of this tick. Only a few *I. hexagonus* and *D. marginatus* specimens were collected, all in April, limiting meaningful comparisons with literature data and preventing reliable assessment of their seasonal activity.

### 4.4. Vector Role

Among the seven tick species recorded, *D. marginatus*, *D. reticulatus*, *I. hexagonus*, *I. ricinus*, and *R. sanguineus* s.l. are known carriers of bacterial, viral, and protozoan pathogens in Croatia [54]. The most common tick-borne pathogens in Croatia include tick-borne encephalitis virus and *Borrelia burgdorferi* s.l., with 400–800 Lyme borreliosis cases annually and 73 TBE cases recorded from 2017 to 2022 in the continental region [55]. Approximately 130,000 cases of Lyme borreliosis occur annually in Europe, with large differences between countries and regions, ranging from less than 1 to more than 360 per 100,000 inhabitants [56]. Recorded cases of tick-borne encephalitis are significantly lesser, with around 4000 cases recorded in 2020 [56]. Given the high vector potential of *I. ricinus* and the overlap between reported human cases and its activity [48], urban green habitats in eastern Croatia may pose a public health risk, highlighting the importance of continued monitoring.

## 5. Conclusions

From March to the end of May 2023 and from mid-March to the end of August 2025, six species of ticks were recorded in green habitats of three cities in eastern Croatia. Most tick species (six of them) were collected in Vinkovci, followed by Osijek and Vukovar with three species each. All collected ticks belong to four genera *Dermacentor*, *Haemaphysalis*, *Ixodes,* and *Rhipicephalus*. In Osijek and Vinkovci, the most abundant species was *I. ricinus*, with a share of 90.89% in Osijek and 70.61% in Vinkovci. In Vukovar, *Rhipicephalus sanguineus* s.l. was the most abundant tick species with 76.36%. The second most abundant species was *H. concinna* in Osijek with 8.94% of all collected ticks, while in Vinkovci it was *R. sanguineus* s.l. with 19.07%. This finding of the species *Rhipicephalus sanguineus* s.l. in Vinkovci and Vukovar represents the first record of this tick species in the area of eastern Croatia. Also, in the green habitats of the city of Vinkovci, *D. reticulatus* was recorded with 5.15%, as well as *I. hexagonus* with 4.12% and *D. marginatus* with 0.51% in the collected tick sample. This study was supplemented by an earlier finding from the city of Vukovar of the species *I. canisuga*, which represents the seventh tick species recorded in the green habitats of the cities studied. In green habitats on the outskirts of Osijek and Vinkovci, significantly more ticks were collected than in the green habitats inside the city. In these green habitats, 99.36% of ticks were collected in Osijek and 62.37% in Vinkovci. These habitats are close and in contact with natural habitats, which is most likely the reason for the high abundance and diversity of ticks in these habitats. In this regard, seven tick species have been recorded in these habitats on the edges of cities, while only two tick species (*I. ricinus* and *R. sanguineus* s.l.) were recorded in green habitats within cities. The high abundance of *I. ricinus* in green habitats of cities may pose a major risk to human health, given that this species is the main vector of *Borrelia burgdorferi* s.l. and TBE virus in Europe.

## Figures and Tables

**Table 1:** Number of hard ticks recorded in the study area.

Tick Species/City	Osijek	Vinkovci	Vukovar	Total	%
*Ixodes ricinus*	569	137	12	718	82.06
*Rhipicephalus sanguineus* s.l	-	37	42	79	9.03
*Haemaphysalis concinna*	56	1	-	57	6.51
*Dermacentor reticulatus*	1	10	-	11	1.26
*Ixodes hexagonus*	-	8	-	8	0.91
*Dermacentor marginatus*	-	1	-	1	0.11
*Ixodes canisuga*	-	-	1	1	0.11
Total	626	194	55	875	99.99

**Table 2:** Number of hard ticks sampled in green habitats in the city of Osijek.

Green Habitats/Tick Species	*I. ricinus*	*H. concinna*	*D. reticulatus*	Total	%
Urban forest in Tvrđavica	410	55	-	465	74.28
Children’s playground in Tvrđavica	93	1	-	94	15.01
Copacabana beach	62	-	-	62	9.90
Forest with a botanical trail along the Drava River	-	-	1	1	0.16
King Tomislav Park	-	-	-	-	-
Meadow near the stadium Gradski vrt	1	-	-	1	0.16
Garo dog park	3	-	-	3	0.48
Toti dog park	-	-	-	-	-
Total	569	56	1	626	99.99

**Table 3:** Number of hard ticks sampled in green habitats in the city of Vukovar.

Green Habitats/Tick Species	*I. ricinus*	*I. canisuga*	*R. sanguineus* s.l.	Total	%
Adica Forest Park	3	-	-	3	5.45
Forest near the Memorial Cemetery (MCVHW)	9	1	-	10	18.18
Mazda Park	-	-	42	42	76.36
Total	12	1	42	55	99.99

**Table 4:** Number of hard ticks sampled in green habitats in the city of Vinkovci.

Green Habitats/Tick Species	*I.* *ricinus*	*R. sanguineus* s.l.	*D.* *reticulatus*	*I.* *hexagonus*	*D.* *marginatus*	*H.* *concinna*	Total	%
Dr. Franjo Tuđman park	3	-	-	-	-	-	3	1.54
Park Lenije	34	6	-	-	-	-	40	20.62
Dog park Lapovci	25	5	-	-	-	-	30	15.46
Promenade along the Bosut River	36	10	4	4	1	-	55	28.35
Sopot picnic area	39	16	6	4	-	1	66	34.02
Total	137	37	10	8	1	1	194	99.99

## Data Availability

The data presented in this study are available on request from the corresponding author.
